# Particulate matter air pollution causes oxidant-mediated increase in gut permeability in mice

**DOI:** 10.1186/1743-8977-8-19

**Published:** 2011-06-09

**Authors:** Ece A Mutlu, Phillip A Engen, Saul Soberanes, Daniela Urich, Christopher B Forsyth, Recep Nigdelioglu, Sergio E Chiarella, Kathryn A Radigan, Angel Gonzalez, Shriram Jakate, Ali Keshavarzian, GR Scott Budinger, Gökhan M Mutlu

**Affiliations:** 1 Department of Medicine, Section of Gastroenterology and Nutrition Rush University Medical College, 1725 W Harrison Street, Chicago, IL, 60612 USA; 2 Department of Medicine, Division of Pulmonary and Critical Care Medicine, Feinberg School of Medicine, Northwestern University, 240 E Huron Street, McGaw M300, Chicago, IL, 60611, USA; 3 Department of Pathology, Rush University Medical College, 1725 W Harrison Street, Chicago, IL, 60612 USA

## Abstract

**Background:**

Exposure to particulate matter (PM) air pollution may be an important environmental factor leading to exacerbations of inflammatory illnesses in the GI tract. PM can gain access to the gastrointestinal (GI) tract via swallowing of air or secretions from the upper airways or mucociliary clearance of inhaled particles.

**Methods:**

We measured PM-induced cell death and mitochondrial ROS generation in Caco-2 cells stably expressing oxidant sensitive GFP localized to mitochondria in the absence or presence of an antioxidant. C57BL/6 mice were exposed to a very high dose of urban PM from Washington, DC (200 μg/mouse) or saline via gastric gavage and small bowel and colonic tissue were harvested for histologic evaluation, and RNA isolation up to 48 hours. Permeability to 4kD dextran was measured at 48 hours.

**Results:**

PM induced mitochondrial ROS generation and cell death in Caco-2 cells. PM also caused oxidant-dependent NF-κB activation, disruption of tight junctions and increased permeability of Caco-2 monolayers. Mice exposed to PM had increased intestinal permeability compared with PBS treated mice. In the small bowel, colocalization of the tight junction protein, ZO-1 was lower in the PM treated animals. In the small bowel and colon, PM exposed mice had higher levels of IL-6 mRNA and reduced levels of ZO-1 mRNA. Increased apoptosis was observed in the colon of PM exposed mice.

**Conclusions:**

Exposure to high doses of urban PM causes oxidant dependent GI epithelial cell death, disruption of tight junction proteins, inflammation and increased permeability in the gut *in vitro *and *in vivo*. These PM-induced changes may contribute to exacerbations of inflammatory disorders of the gut.

## Background

In human populations, investigators have associated PM exposure with an increasing number of adverse health outcomes including all-cause mortality, cardiovascular mortality, accelerated atherosclerosis in postmenopausal women, loss of lung function in healthy adults, impaired lung development in children, exacerbations of obstructive lung disease, pneumonia and increased risk of venous thromboembolism [1-11]. Recently, Kaplan et al reported that individuals younger than 23 years were more likely to be diagnosed with Crohn's disease if they lived in regions with higher NO_2 _concentrations [12], a marker of traffic related pollution that correlates with the levels of PM and an independent group of investigators reported that exposure to PM was associated with hospitalizations in patients with inflammatory bowel disease [13]. PM exposure has also been reported to increase the risk of appendicitis,[14] the development of gastroenteritis in children [15] and colorectal cancer [16-19].

Many of the adverse health consequences of PM are thought to be caused by the ability of PM to induce cellular oxidative stress, which activates signaling pathways that contribute to cytokine release and barrier dysfunction [20]. As the bulk of PM enters the body by inhalation, investigators have used radiolabeled particles to examine the fate of inhaled particles in rodents [21]. They found that the overwhelming majority of the particles (> 95%) are taken up by alveolar macrophages, brought to the oropharynx by mucociliary clearance and excreted in the feces [21].

PM may also enter the GI tract directly through swallowed air or through the ingestion of foods such as vegetables and fruits coated with PM. These findings suggest that the steady state concentrations of PM in the GI tract may be similar to those encountered in the lung. In this paper, we sought to test the hypothesis that PM exposure can induce oxidant dependent epithelial dysfunction of the GI tract in mice. We tested our hypothesis in cell based systems and in mice using a very high dose of a well characterized urban PM.

## Methods

### Particulate Matter (PM)

We used an urban PM collected from ambient air in Washington, DC (National Institute of Standards and Technology Standard Reference Material, SRM 1649a). The characteristics of PM have been previously described [22,23].

### Animals and administration of PM via gavage

The protocol for the use of mice was approved by the Animal Care and Use Committee at Northwestern University and Rush University in Chicago, IL. We used eight to twelve weeks old, (20-25 g), male, C57BL/6 mice (Jackson Laboratories). Mice were anesthetized with isoflurane 2-3%. After adequate anesthesia is achieved, the esophagus was intubated using a mouse gastric gavage tube and 200 μg of PM suspended in sterile PBS or PBS only (control) was administered via gavage tube followed by 100 μl of air to clear the liquid in the gavage tube. Particulate matter does not dispersed well in aqueous solutions because it forms aggregates in PBS or in culture media within several minutes. Therefore, PM in PBS was vortexed immediately prior to instillation [24]. After treatment, the gavage tube was removed gently and the animals were placed in their cages until harvesting of stomach, small bowel and colon at 24 and 48 hours. Permeability experiments were also performed at 48 hours.

### Histology

At 24 or 48 hours after treatment with PM, mice were euthanized for collection of stomach, small bowel and colon tissue. After harvesting, the tissues were fixed and paraffin embedded and 5-μm sections were stained with hematoxylin/eosin.

### TUNEL staining of colonic sections

End labeling of exposed 3'-OH ends of DNA fragments in paraffin-embedded tissue was done utilizing the TUNEL AP In Situ Cell Death Detection Kit (Roche Diagnostics Corp.) according to the manufacturer's directions. After staining, 20 fields of colonic sections (400x) were randomly chosen and nuclei and total nuclei were counted using an automated program (Image J) [25].

### Assessment of intestinal permeability in mice

GI permeability was measured based on the intestinal permeability towards 4000Da-fluorescent-dextran- FITC (DX-4000-FITC) (FD4000 Sigma-Aldrich, St. Louis, Mo, USA) as described [26,27]. Briefly, mice were given DX-4000-FITC by gavage (500 mg/kg body-weight, 125 mg/ml). After 1 hour, 120 μl of blood were collected via right atrial puncture. The blood was centrifuged at 4°C, 12000×g for 3 min. Plasma was diluted in an equal volume of PBS (pH 7.4) and analyzed for DX-4000-FITC concentration with a fluorescence spectrophotometer at the excitation wavelength of 485 nm and the emission wavelength of 535 nm. Standard curves were obtained by diluting FITC-dextran in non-treated plasma diluted with PBS (1:3 v/v).

### Cell culture

Caco-2 cells (a human colon adenocarcinoma cell line) (American Type Culture Collection, Manassas, VA) were chosen for our studies because they form monolayers that morphologically resemble intestinal cells with defined apical brush borders. Cells were cultured in Dulbecco's minimum essential medium at 37°C, 5% CO_2_, 100% relative humidity. Cells were exposed to PM diluted in media or control vehicle (media). To ensure equal dispersion, the PM solution was vortexed with the media before application to the cells. For barrier function experiments, Caco-2 cells were split at a ratio of 1:2 and seeded at a density of 200,000 cells/cm^2 ^into 0.4 μM Biocoat Collagen I Cell Culture Inserts (0.3 cm^2 ^growth surface; Becton Dickinson Labware, Bedford, MA) and experiments were performed at least 3 weeks post-confluence. The confluence of Caco-2 monolayers was confirmed with transepithelial electrical resistance (TEER) measurements as described below [28,29]. Only monolayers of cells with TEER values greater than 250 Ω × cm^2 ^(after subtraction of the bare filter inserts [100-150 Ω × cm^2^]) were used for the experiments as previously described by Turner et al and other investigators [28,30-33]. The confluent monolayers were exposed to PM suspended in media or vehicle control (media alone). To ensure equal dispersion, the PM solution was vortexed with the media before application to the cells.

### Measurement of transepithelial electrical resistance (TEER)

Transepithelial electrical resistance (TEER) was determined in polarized Caco-2 cell monolayers, cultured in 6-well plates, using a Millicell-ERS Resistance System (Millipore, Bedford, MA) that includes a dual electrode volt-ohm-meter. TEER was calculated as TEER = (*R*_m_-*R*_i_) × *A*, where *R*_m _is transmembrane resistance; *R*_i_, intrinsic resistance of a cell-free media; and *A*, the surface area of the membrane in cm^2 ^[28,29,34]. The TEER measurements were performed in serum free media.

### Measurement of the apparent permeability coefficient (Papp) of fluorescein sulfonic acid (FSA)

Barrier permeability was determined using Caco-2 cells grown to confluence on 6.5-mm/0.4 μm 24-well tissue culture plate inserts (Transwell, Corning) as we have previously described [35,36]. The measurements were performed in serum free media. Permeability of insert Caco-2 monolayers was evaluated by measurements of the apparent permeability coefficient (Papp) of the fluorescent marker fluorescein-5-(and-6)-sulfonic acid trisodium salt (FSA, 200 μg/ml, 478 Da) (Invitrogen) in the apical to basolateral direction [33]. Fluorescent signals from samples were measured using a fluorescence multiplate reader (FSA excitation, 485 nm; FSA emission, 530 nm). The Papp was calculated by the following formula: Papp (cm/sec) = (dQ/dt)/(Co × A), where dQ/dt is the permeability rate (mol/s); Co is the initial concentration in the donor compartment (mol/ml) and A is the surface area of the monolayer (0.33 cm^2^).

### Assessment of cell death

Cell death was assessed using a commercially available photometric immunoassay that detects histone-associated DNA fragments (Roche Diagnostics, Indianapolis, IN) according to the manufacturer's directions, as we have previously described [37].

### Generation of Caco-2 cells stably expressing mitochondrially localized oxidant sensitive GFP (mito-Ro-GFP) probes

An adenoviral vector encoding Ro-GFP with a mitochondrial localization sequence (mito-Ro-GFP) was generated as described previously and commercially amplified (ViraQuest, Iowa City, IA) [37,38]. A lentiviral vector encoding the mito-Ro-GFP probe with a mitochondrial localization sequence was created using the ViraPower lentiviral transformation kit according to the manufacturer's directions (Invitrogen) as previously described [37]. Expression of the probe was confirmed by examining the cells using fluorescence microscopy. Appropriate localization of the probe to the mitochondria was confirmed by co-staining with the mitochondrial probe MitoTracker (10 μM) (Invitrogen, Carlsbad, CA).

### Generation of Caco-2 cells lacking mitochondrial DNA (ρ^0 ^Caco-2 cells)

The ρ^0 ^Caco-2 cells were generated by incubating Caco-2 cells in medium containing ethidium bromide (100 ng/ml), sodium pyruvate (1 mm), and uridine (100 μg/ml) for 3-5 weeks. The lack of DNA encoding cytochrome oxidase subunit IV was confirmed by PCR using the primer sequences described previously [37,39,40]. Cells depleted of mitochondrial DNA, or ρ^0 ^cells, lack 13 proteins critical for normal electron transport. Although ρ^0 ^cells contain petite mitochondria, they cannot support normal oxidative phosphorylation and must survive and replicate using ATP derived solely from glycolysis. Without a functional electron transport chain, ρ^0 ^cells cannot generate ATP from electron transport and their mitochondria are incapable of generating reactive oxygen species [40,41].

### Measurement of Reactive Oxygen Species

We employed an oxidant-sensitive ratiometric probe (Ro-GFP) that was originally described by Hanson and co-workers who validated its responsiveness to a variety of intracellular oxidants both ex vivo and in living cells [42,43]. We generated Caco-2 cells stably expressing the mito-Ro-GFP probe and measured the oxidation state of the probe using flow cytometry as we have previously described [37]. Briefly, the cells were removed from the plate using trypsin, and equal aliquots of the resulting suspension were transferred to tubes containing medium alone or medium containing 1 mM dithiothreitol or 1 mM *t*-butyl hydroperoxide (t-BOOH). After 10 min, the ratio of fluorescence (emission of 535 nm) at excitations of 405 and 488 nm was measured in 5,000 cells/condition using a DakoCytomation CyAn high speed multilaser droplet cell sorter. The oxidation state of the cells was calculated as follows: (value after treatment - value after dithiothreitol (completely reduced state))/(value after t-BOOH (completely oxided state) - value after dithiothreitol (completely reduced state)) [37,38]. In some experiments, we also treated cells with EUK-134 (20 μM) (Cayman Chemicals, Ann Arbor, MI), which is a combined superoxide dismutase and catalase mimetic that has been shown to prolong the lifespan of the nematode Caenorhabditis elegans and to prevent alveolar epithelial cell death during injurious stimuli [44,45].

### Immunoblotting determination of nitration and oxidation of tubulin to assess cytoskeleton

Oxidation and nitration of the tubulin backbone of microtubules were assessed by measuring protein carbonyl and nitrotyrosine formation, respectively [46]. Carbonylation and nitrotyrosination of tubulin were determined in a similar manner as the quantitative blotting of tubulin. To avoid unwanted oxidation of tubulin samples, all buffers contained 0.5 mM dithiothreitol and 20 mM 4,5-dihydroxy-1,3-benzene sulfonic acid (Sigma).

### Immunofluorescent staining and high-resolution laser scanning confocal microscopy of the actin cytoskeleton

Integrity of actin cytoskeleton integrity in monolayers of Caco-2 cells was determined as previously described [47]. Cells were subsequently processed by incubation with fluorescein isothiocyanate (FITC)-conjugated phalloidin (specific for F-actin staining, Sigma, St. Louis, MO), 1:40 dilution for 1 h at 37°C. Desired areas of monolayers were processed using the image processing with Zeiss Axiovision and NIH Image J software.

### Analysis of NF-κB activation

NF-κB (p65 and p50 subunit) activation was assessed by ELISA as we have previously described [35,48]. The NF-κB activity test is based on a validated ELISA principle in which NF-κB is captured by a double stranded oligonucleotide probe containing the consensus-binding sequence for either NF-κB p65 or p50 subunits [49]. The results were quantified by a chromogenic reaction, which was then read for absorbance at 450 nm by a Seivers NOA 280 microplate analyzer (Sievers, CO) [49].

### Immunofluorescence staining of zonula occludens-1 (ZO-1) and occludin for tight junction morphology

Immunofluorescence staining for ZO-1 and occludin was performed in Caco-2 cells and fresh frozen mouse small bowel tissue sections using primary anti-ZO-1 antibody (Zymed) (1:200 dilution) or for anti-occludin antibody (Zymed) (1:100 dilution) for1 hour at 37°C followed by incubation with a secondary antibody (fluorescein isothiocyanate-conjugated goat anti-mouse; Sigma-Aldrich, St. Louis, MO; 1:50 dilution) for 1 hour at room temperature. Imaged areas of monolayers were processed using Zeiss Axiovision and NIH Image J image processing software.

### Evaluation of tight junction disruption

Caco-2 cells were stained for ZO-1 and the slides were examined for their overall morphology, orientation, and disruption as we previously described [35,50]. To avoid bias, the slides were coded so that the examiner was blinded to the experimental protocol. After the scoring was complete, the slides were decoded for analysis. Briefly, the "ZO-1 integrity" in Caco-2 cells was considered abnormal or injured based on one or more of the following criteria: fragmentation, kinking, disruption of actin cortex, or detachment from membrane areas (areas of cell-to-cell contact). We examined 200 cells per slide (well) by LSCM microscopy, and the percentage (%) of cells displaying normal ZO-1 or abnormal ZO-1 was determined (e.g., 90 cells [displaying injured ZO-1] divided by 200 is equal to 45% abnormal ZO-1 or 55% normal ZO-1). Overall, at least 1,200 cells per group (200 × 6 slides) were examined in four different fields by LSCM and the percentage of cells displaying normal ZO-1 was then determined per treatment group.

### Statistics

Differences between groups were explored using analysis of variance. When the analysis of variance indicated a significant difference, individual differences were explored using t tests with a Dunnett correction for multiple comparisons against control conditions. All of the analyses were performed using GraphPad Prism version 4.00 for Windows (GraphPad Software, San Diego, CA).

## Results

### Particulate matter induces a dose-dependent ROS generation and cell death in colonic epithelial cells

**Figure 1:**
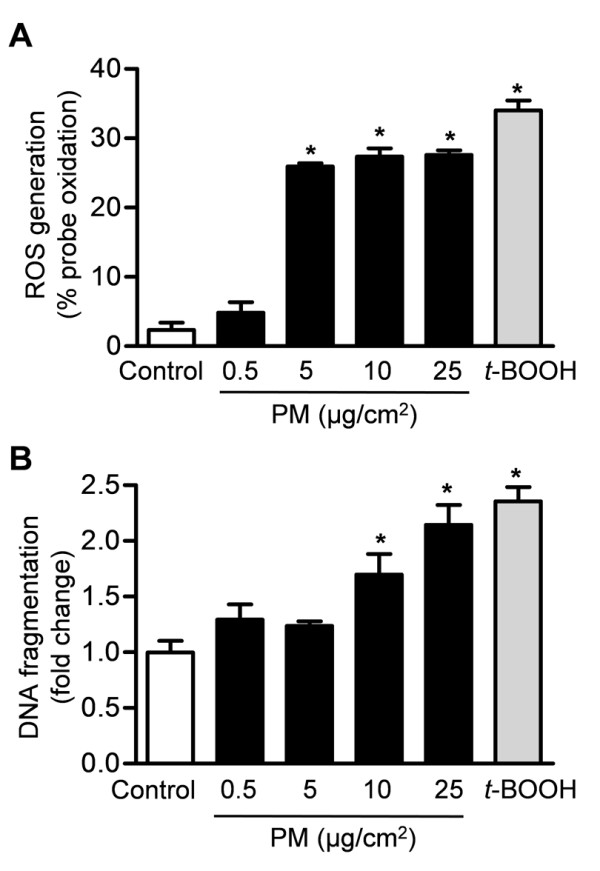
**Particulate matter induces dose-dependent ROS generation and cell death in colonic epithelial cells**. **(A) **Caco-2 cells stably expressing an oxidant-sensitive GFP probe (mito-Ro-GFP, lentivirus) were treated with control vehicle (media) or different doses of PM. The percentage oxidation of the probe was measured using flow cytometry 4 hours later. The *t*-butyl hydroperoxide (*t*-BOOH) was used as a positive control. (**B) **Caco-2 cells were treated as described above and cell death (DNA fragmentation via enzyme-linked immunosorbent assay) was measured 24 hours later. (*P < 0.05 PM compared with PBS, n ≥ 4).

We previously reported that exposure of alveolar epithelial cells to PM_10 _collected from Düsseldorf, Germany induced the generation of ROS [51]. To determine whether PM can induce ROS generation in the GI tract epithelia, we treated Caco-2 cells stably expressing mitochondrial oxidant sensitive GFP (mito-Ro-GFP) with vehicle (media) or different concentrations of urban PM from Washington, D.C. Four hours later, we assessed PM-induced ROS production by measuring the oxidation of the probe. Treatment with *t*-butyl hydroperoxide was used as a positive control. Exposure of Caco-2 cells to PM caused a dose-dependent ROS generation from the mitochondria (Figure 1A). To determine whether PM can induce cell death in the GI tract epithelia, we treated Caco-2 cells with vehicle (media) or different concentrations of PM for 24 hours and measured apoptotic cell death (DNA fragmentation). Exposure of Caco-2 cells to PM caused a dose-dependent increase in the percentage of TUNEL positive cells (Figure 1B).

### The generation of mitochondrial ROS is required for PM-induced cell death in colonic epithelial cells

**Figure 2:**
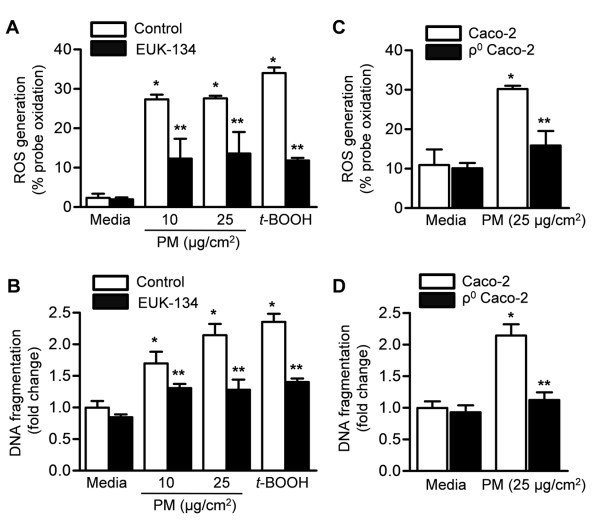
**The generation of mitochondrial ROS is required for PM-induced cell death in colonic epithelial cells**. **(A) **ROS production and **(B) **cell death were measured 4 and 24 hours, respectively, after treatment of Caco-2 cells stably expressing mitochondrial oxidant sensitive GFP (mito-Ro-GFP) with control (media) or PM in the absence or presence of the combined superoxide dismutase and catalase mimetic, EUK -134 (20 μM). **(C) **ROS production and **(D) **cell death were measured at 4 and 24 hours, respectively, after treatment of wild-type Caco-2 cells and Caco-2 cells lacking mitochondrial DNA (ρ^0 ^CaCo-2 cells) with vehicle (media) or PM. The *t*-butyl hydroperoxide (*t*-BOOH) was used as a positive control. (P < 0.05 *PM compared with PBS, **EUK-134 compared with vehicle, n ≥ 4).

We treated Caco-2 cells stably expressing mitochondrial oxidant sensitive GFP (mito-Ro-GFP) with vehicle (media) or different concentrations of PM from Washington, D.C in the absence or presence of the combined superoxide dismutase and catalase mimetic, EUK -134 (20 μM), which was added to the media 1 hour before exposure. We then measured oxidation of the mitochondrially localized probe 4 hours later and apoptotic cell death 24 hours later. Both PM-induced ROS generation and cell death were attenuated in cells treated with (EUK-134) therapy (Figure 2A,B).

To determine whether mitochondrially generated ROS are required for PM-induced cell death, we generated Caco-2 cells lacking mitochondrial DNA (ρ^0 ^Caco-2 cells). Wild type and ρ^0 ^Caco-2 cells were infected with an adenovirus encoding the mito-Ro-GFP, and the oxidation of the probe was measured at 4 hours after treatment with PM (25 μg/cm^2^) suspended in media or control vehicle (media) using flow cytometry. Compared to wild-type cells, ρ^0 ^Caco-2 cells produced minimal ROS in response to PM (Figure 2C). We treated wild-type and ρ^0 ^Caco-2 cells with PM and measured cell death 24 hours later. Compared with wild-type cells, PM-induced cell death was attenuated in the ρ^0 ^Caco-2 cells (Figure 2D).

### Exposure to PM causes oxidant-dependent NF-κB activation and increased permeability in Caco-2 cell monolayers

**Figure 3:**
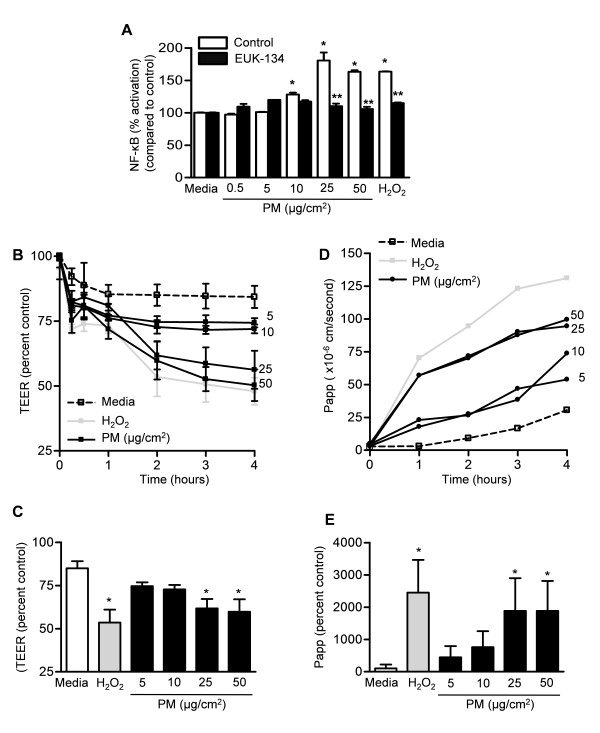
**Exposure of Caco-2 cells to PM causes oxidant-dependent NF-κB activation and increased permeability**. **(A) **NF-κB activation was measured in Caco-2 cells treated with vehicle (PBS) or different doses of PM in the absence or the presence of the combined superoxide dismutase and catalase mimetic, EUK -134 (20 μM). NF-κB activation was presented as % activation compared to H_2_O_2 _(positive control). To evaluate the permeability of the Caco-2 monolayer, cells were treated with either vehicle (media) or different concentrations of PM and **(B) **transepithelial electrical resistance (TEER) was measured over time. **(C) **TEER values compared to control (as percent control) at 2 hours are shown. **(D) **We also measured the apparent permeability co-efficient (Papp) of fluorescein sulfonic acid (FSA) in the apical to basolateral direction across the Caco-2 cells. **(E) **Percent change in Papp values at 2 hours is shown. (P < 0.05 *PM compared with PBS, **EUK-134 compared with vehicle, n ≥ 4).

As oxidant stress in the gut oxidation has been associated with activation of the nuclear transcription factor, NF-κB [52], we measured NF-κB in Caco-2 cells exposed to either PM or vehicle (PBS). Exposure to PM caused a dose-dependent increase in NF-κB activity, which was prevented by, EUK-134 (Figure 3A).

Intestinal barrier disruption has been implicated in several intestinal and systemic disorders and has been associated with ROS generation, NF-κB activation and cell death [50,53]. We treated Caco-2 cells with either vehicle (media) or different concentrations of PM and assessed the permeability of the monolayer by measuring the transepithelial electrical resistance (TEER) (Figure 3B) and the apparent permeability coefficient (Papp) of fluorescein sulfonic acid (FSA) in the apical to basolateral direction (Figure 3C and 3D). Exposure to PM caused an increase in permeability (Figure 3).

### Exposure to particulate matter causes nitration and oxidation injury and disruption of the microtubule cytoskeleton of intestinal cell monolayers

**Figure 4:**
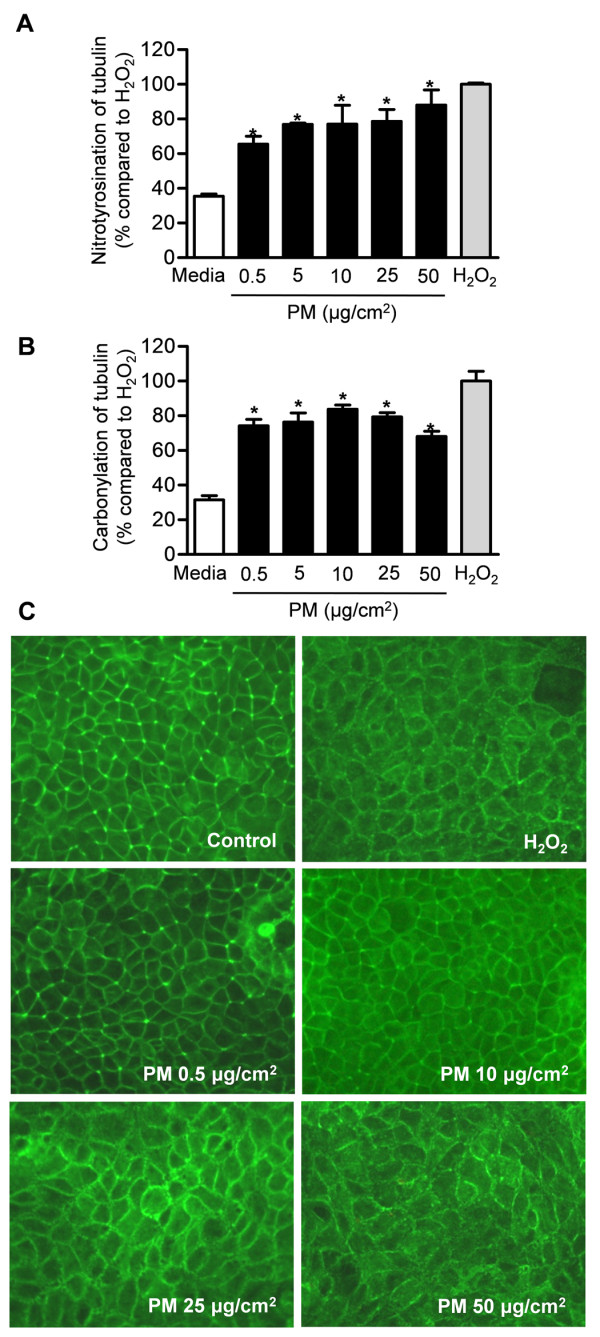
**Exposure to particulate matter causes nitration and oxidation of tubulin and disruption of tight junctions in intestinal cell monolayers**. The effect of PM on tubulin **(A) **nitration (nitrotyrosination) and **(B) **oxidation (carbonylation) in Caco-2 cell monolayers are shown. H_2_O_2 _(0.5 mM) was used as a positive control. **(C) **Images from high-resolution laser scanning confocal microscopic evaluation of intestinal cell monolayers stained for F-actin are shown.

We assessed the effect of PM induced ROS generation on the cytoskeleton by measuring the amount of nitration (nitrotyrosination) and oxidation (carbonylation) of tubulin in Caco-2 cells as previously described [46]. Oxidant (H_2_O_2_, 0.5 mM) alone resulted in substantial levels of nitration (nitro and oxidation of the tubulin cytoskeleton (Figure 4A and 4B)). Exposure to Caco-2 cells to PM caused nitration and oxidation of the tubulin cytoskeleton, which was evident even at the lowest dose of PM (0.5 μg/cm^2^). High-resolution laser scanning confocal microscopic evaluation of Caco-2 monolayers confirmed that intestinal cells exhibited abnormal architecture of the microtubule cytoskeleton after treatment with PM. The changes seen at highest dose of PM (50 μg/cm^2^) were similar to those seen from cell monolayers that are challenged with H_2_O_2 _(Figure 4C).

### Exposure of Caco-2 cells to particulate matter causes disruption of tight junctions

**Figure 5:**
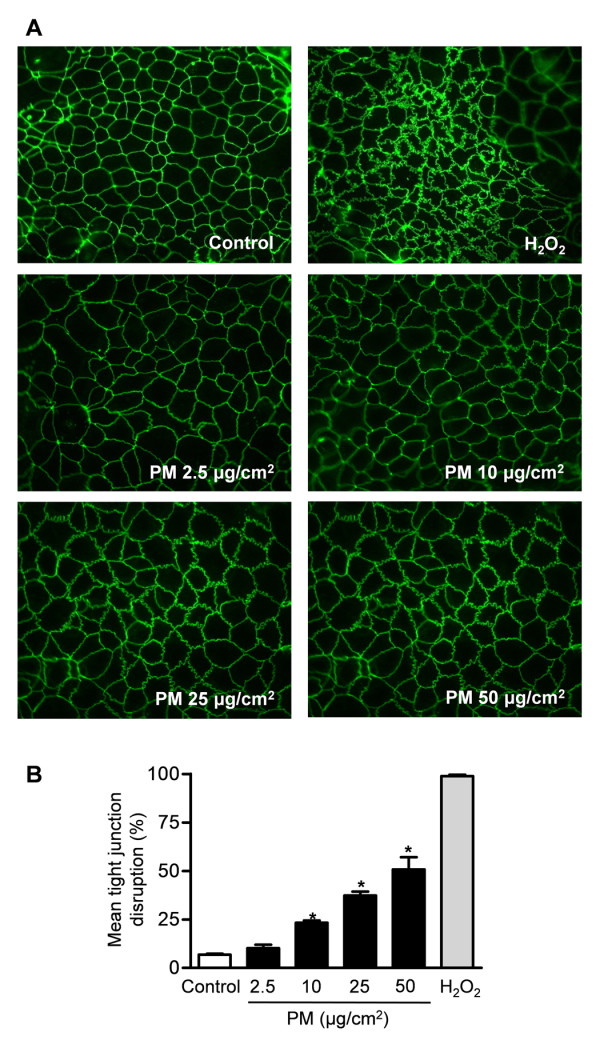
**Exposure of monolayers of Caco-2 cells to PM causes increased permeability and disruption of tight junctions**. Caco-2 cells were treated with either PBS (vehicle) or different concentrations of PM and we **(A) **stained the cell monolayers for ZO-1, a tight junction protein and **(B) **measured mean tight junction disruption Intestinal sections from the animals were subjected to a blinded analysis of tight junction disruption. Representative images from 4 independent experiments are shown and each bar represents the mean of 20 observations.

To determine whether PM-induced increased permeability in monolayers of Caco-2 cells is associated with changes in their tight junctions, we stained monolayers of Caco-2 cells for ZO-1, a tight junction protein. Caco-2 cells exposed to PM had discontinuity and irregularity of ZO-1 distribution at the intercellular junctions suggesting the loss of tension in intestinal cells. ZO-1 was rearranged from an uninterrupted band along cell borders into radially oriented sporadic aggregates (Figure 5A). The severity of tight junction disruption estimated from blinded analysis of ZO-1 stained sections for their overall morphology, orientation, and disruption was increased in PM compared with control treated Caco-2 cells (Figure 5B).

### Exposure to PM causes increased gut permeability and inflammation and colonic epithelial cell death in mice

**Figure 6:**
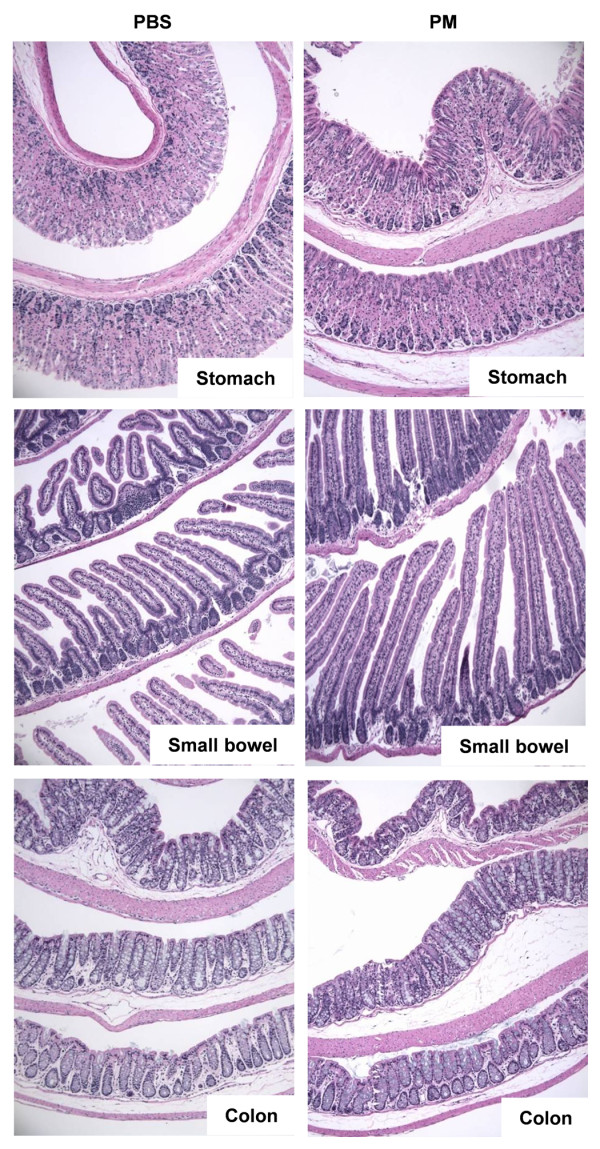
**Exposure to PM does not cause histologic changes in gastric, small bowel and colonic mucosa at 24 or 48 hours**. Wild-type cells were treated with either PBS (vehicle) or PM via gastric gavage and histology (H&E staining) of gastric, small bowel and colonic tissue was evaluated 48 hours after exposure. Representative photomicroographs from a total of 5 animals/treatment group are shown (x100 magnification).

**Figure 7:**
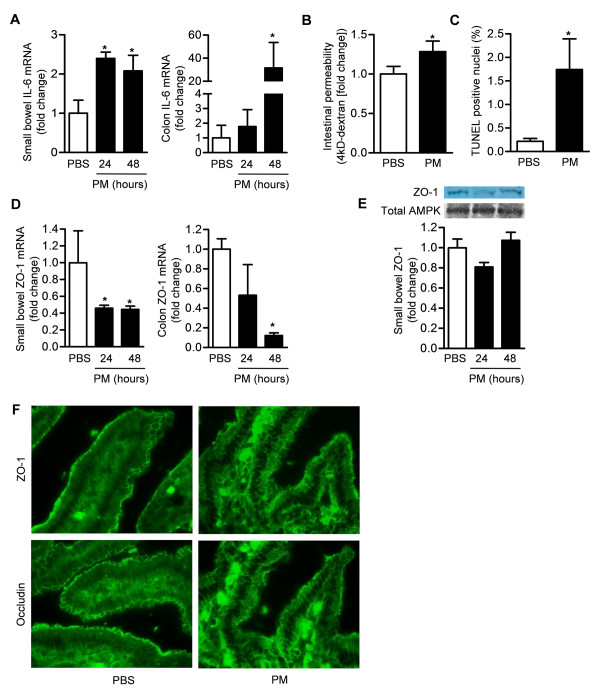
**Exposure to PM causes increased gut permeability and inflammation and colonic epithelial cell death in mice**. Wild-type mice were treated with either PBS (vehicle) or PM via gastric gavage for measurement of **(A) **IL-6 mRNA (qRT-PCR) in small bowel and colonic homogenates (24 and 48 hours after treatment) (n = 6/treatment group), **(B) **intestinal permeability to 4kD dextran (48 hours after treatment) (n = 5/treatment group), **(C) **cell death (TUNEL-positive nuclei) in the colon (48 hours after treatment) (n = 4/treatment group), **(D) **ZO-1 mRNA (qRT-PCR) in the small bowel and colon (n = 4/treatment group) and **(E) **ZO-1 protein expression in the small bowel (24 and 48 hours after treatment) (n = 4/treatment group) and **(F) **tight junction disruption (ZO-1 and occludin localization) in the small bowel by immunofluorescence (48 hours after treatment) (Representative images, n = 4/treatment/group). (P < 0.05 *PM compared with PBS).

To determine whether PM exposure affects GI epithelia *in vivo*, we treated mice with PM administered via gavage and harvested the stomach and colon 48 hours later. Exposure to PM did not cause any detectable histologic changes in the GI tract (Figure 6). However, compared to PBS treated animals, mice exposed to PM exhibited an increase in IL-6 mRNA in the small bowel and colon suggesting that PM induces an inflammatory response in the gut (Figure 7A).

We measured the permeability of the gut permeability to a 4kD dextran (Figure 7B) 48 hours after the administration of PM or PBS via gavage. PM exposure was associated with a significant increase in permeability. We then counted the number of TUNEL-positive nuclei observed in colonic sections obtained 48 hours after treatment with PM or PBS. Treatment with PM was associated with an increase in the number of TUNEL positive nuclei (Figure 7C). Forty-eight hours after treatment with PM, we observed a significant reduction in the mRNA encoding ZO-1 measured by qRT-PCR in the small bowel and colon (Figure 7D). The ZO-1 protein expression in the small bowel did not change significantly after PM treatment (Figure 7E). Examination of ZO-1 and occludin stained sections of small bowel tissue was altered in the PM compared with the PBS treated mice (Figure 7F).

## Discussion

Exposure to air pollution is associated with well-known health effects on the cardiovascular and respiratory systems [54-56]. While less known, there also are several reports linking exposure to air pollution to adverse health effects in the GI tract [12-19]. Consistent with our findings in lung epithelial cells, we observed that Caco-2 cells exposed to PM generated ROS from the mitochondria, which were required for the activation of NF-κB and the development of apoptotic cell death *in vitro*. In confluent monolayers of Caco-2 cells, we found that PM reduced the TER of the monolayer and caused disruption of the architecture of the tight junctions. In mice exposed to PM by oral gavage, we found evidence of increased IL-6 transcription, disruption of small bowel tight junctions, increased numbers of TUNEL positive nuclei and increased gut permeability to FITC labeled dextran.

We found that colonic epithelial cells generate significant amounts of mitochondrially derived ROS and undergo apoptosis in response to PM exposure *in vitro*. Cells depleted of mitochondrial DNA (ρ^0 ^Caco-2 cells) were resistant to PM induced apoptosis. ρ^0 ^cells lack the 13 proteins encoded in the mitochondrial genome, which are required for normal electron transport. As a result, these cells are deficient in oxidative phosphorylation and cannot produce mitochondrial ROS. However, as most of the mitochondrial proteome is encoded by nuclear genes, these cells still contain mitochondria, maintain a mitochondrial membrane potential supported by glycolysis and undergo apoptosis normally. We reported a similar dependence of PM-induced signaling and apoptosis in alveolar epithelial cells and other investigators have highlighted the importance of mitochondrially derived ROS in the cellular response to PM [22,37,51]. Inflammation the GI tract during diseases such as inflammatory bowel disease is associated with increased local ROS generation and activation of NF-κB[57-61]. We observed that the administration of PM to Caco-2 cells resulted in the oxidant dependent activation of NF-κB. The importance of these findings is highlighted by our observation that the administration of PM by oral gavage increased the transcription of an NF-κB target gene IL-6 in the small bowel and colon.

We observed that the administration of PM to Caco-2 monolayers reduced the transepithelial resistance of the monolayers and caused a disruption in the organization of the tight junctions. *In vivo*, the administration of PM was associated with decreased transcription of the tight junction proteins ZO-1 and occludin and histologic evidence of alterations in tight junction structure. There was also an increase in the number of apoptotic cells in the small bowel from these animals. Our data do not allow us to determine whether PM-induced alterations in tight junctions, apoptosis or other mechanisms are responsible for the resulting increase in gut permeability. However, this finding is important as chances in gut permeability are thought to play a pathogenic role in many gastrointestinal disorders including alcoholic liver disease, inflammatory bowel disease and celiac disease [57,61,62].

While the lungs are the primary organs exposed to ambient PM; a significant portion of PM exposure can be deposited in the upper airway and also get access to the GI tract via direct entrance due to swallowing of air or indirectly via swallowing of the PM deposited in the upper airway. Furthermore, most of the inhaled PM is transported via mucociliary system from the lung to the upper airway and then swallowed. Our data support the recent reports, which showed an epidemiologic link between acute, short-term exposure to air pollution and inflammation in the gut. We speculate that PM-induced ROS generation, NF-κB activation and activation of inflammation may be responsible for exacerbations of inflammatory conditions in the gut such as inflammatory bowel disease.

Urban PM is a complex mixture of solid and particles that primarily are formed during the combustion of fossil fuels [63,62]. As the carbon core of the particle cools, organic components (including quinones) and transition metals (including iron and vanadium) condense on the surface of the particles. The resulting particles are composed of a carbonaceous core, organic components, sulfate, nitrate, ammonium, crustal material and transition metals such as iron or vanadium. Organic constituents in the particles, particularly quinones, can undergo single electron transfer reactions with intracellular electron acceptors, creating the opportunity for the transfer of an electron to molecular oxygen to form a superoxide radical. In the presence of a transition metal, the superoxide anion can be converted to a hydroxyl radical through Fenton chemistry [63-67]. In addition, sulfates and nitrates may combine with water to form acids which may modulate their toxicity.

An important limitation of our study is the dose of PM that we employed for our *in vivo *experiments. PM enters the lung via inhalation and larger particles are deposited in the oropharynx and large airways, while smaller particles gain access to the distal airspaces. A very small fraction of PM may gains access to the systemic circulation and affect systemic organs including the gut. Semmler-Benke followed the fate of iridium labeled carbon black nanoparticles administered via inhalation to rats for 6 months after the exposure. Most of the particles (> 90%) were taken up by alveolar macrophages, cleared by mucociliary mechanisms and excreted in the feces [21]. Because the GI tract is also exposed to PM deposited in the oropharynx, PM that has sedimented on unwashed fruits and vegetables and PM contained in swallowed air, the total dose of PM to which the gut is exposed is likely at least as large as that seen by the lung. However, the more rapid transit time of the mucous layer over the gut compared with the lung mucosa may decrease the concentration of PM to which the gut is exposed at any given time. Furthermore, the administration of PM may alter the transit time of material through the gut.

In 2002, the U.S. EPA reported a range of maximal city PM concentrations between 26 and 534 μg/m^3 ^[54]. Many large cities in the world have much higher levels of PM with annual averages of 200 to 600 μg/m^3 ^and peak concentrations frequently exceeding 1000 μg/m^3 ^[68]. Using the highest value in U.S. and assuming a minute ventilation of 6 L/min (~8.6 m^3 ^over 24 hours) for a healthy adult at rest, the total dose of PM inhaled over 24 hours would be 4,614 μg. However, in other "mega cities" of the world, the daily inhaled dose of particles may be as high as 20,000 μg [68,69]. Exposure of a mouse (with a minute ventilation of 35-50 ml/min) to a daily dose of 200 μg of PM corresponds to 24-30 mg of PM exposure for a human [70]. This is about 6-8 times higher than a US adult will be exposed in even the most polluted city. Further study is required to determine whether PM induces similar effects on GI function at lower levels of exposure.

## Conclusions

Exposure to high doses of PM causes oxidant dependent GI epithelial cell death, disruption of tight junctional proteins, inflammation and increased permeability in the gut *in vitro *and *in vivo*. These PM-induced changes may be responsible for exacerbations of inflammatory disorders of the gut. More studies are warranted to better understand the health effects of air pollution in the GI tract.

## Abbreviations

GFP: green fluorescent protein; GI: gastrointestinal; FITC: fluorescein isothiocyanate; PBS: phosphate buffered saline; PM: particulate matter; qRT-PCR: quantitative real-time PCR; ROS: reactive oxygen species; *t*-BOOH: *t*-butyl hydroperoxide; ZO-1: zonula occludens-1.

## Competing interests

The authors declare that they have no competing interests.

## Authors' contributions

EAM, CF, AK, GRSB and GMM: Designed research, analyzed and interpreted data and wrote the manuscript. PE, SS, DU, CF, RN, SEC, KAR, AG and GMM performed research, analyzed data and reviewed the manuscript. SJ, EAM and AK reviewed histology slides.

All authors have read and approved the final manuscript.

## References

[B1] Downs SH Schindler C Liu LJ Keidel D Bayer-Oglesby L Brutsche MH Gerbase MW Keller R Kunzli N Leuenberger P Probst-Hensch NM Tschopp JM Zellweger JP Rochat T Schwartz J Ackermann-Liebrich U SAPALDIA Team Reduced exposure to PM10 and attenuated age-related decline in lung function N Engl J Med 2007 357 2338 2347 10.1056/NEJMoa073625 18057336

[B2] Gauderman WJ Avol E Gilliland F Vora H Thomas D Berhane K McConnell R Kuenzli N Lurmann F Rappaport E Margolis H Bates D Peters J The Effect of Air Pollution on Lung Development from 10 to 18 Years of Age N Engl J Med 2004 351 1057 1067 10.1056/NEJMoa040610 15356303

[B3] Krewski D Burnett RT Goldberg MS Hoover K Siemiatycki J Abrahamowicz M White WH Validation of the Harvard Six Cities Study of particulate air pollution and mortality N Engl J Med 2004 350 198 199 10.1056/NEJM200401083500225 14711928

[B4] McCreanor J Cullinan P Nieuwenhuijsen MJ Stewart-Evans J Malliarou E Jarup L Harrington R Svartengren M Han IK Ohman-Strickland P Chung KF Zhang J Respiratory effects of exposure to diesel traffic in persons with asthma N Engl J Med 2007 357 2348 2358 10.1056/NEJMoa071535 18057337

[B5] Miller KA Siscovick DS Sheppard L Shepherd K Sullivan JH Anderson GL Kaufman JD Long-Term Exposure to Air Pollution and Incidence of Cardiovascular Events in Women N Engl J Med 2007 356 447 458 10.1056/NEJMoa054409 17267905

[B6] Mills NL Tornqvist H Gonzalez MC Vink E Robinson SD Soderberg S Boon NA Donaldson K Sandstrom T Blomberg A Newby DE Ischemic and Thrombotic Effects of Dilute Diesel-Exhaust Inhalation in Men with Coronary Heart Disease N Engl J Med 2007 357 1075 1082 10.1056/NEJMoa066314 17855668

[B7] Pope CA III Ezzati M Dockery DW Fine-Particulate Air Pollution and Life Expectancy in the United States N Engl J Med 2009 360 376 386 10.1056/NEJMsa0805646 19164188 PMC3382057

[B8] Neupane B Jerrett M Burnett RT Marrie T Arain A Loeb M Long-Term Exposure to Ambient Air Pollution and Risk of Hospitalization with Community-acquired Pneumonia in Older Adults Am J Respir Crit Care Med 2010 181 47 53 10.1164/rccm.200901-0160OC 19797763

[B9] Baccarelli A Martinelli I Zanobetti A Grillo P Hou LF Bertazzi PA Mannucci PM Schwartz J Exposure to Particulate Air Pollution and Risk of Deep Vein Thrombosis Arch Intern Med 2008 168 920 927 10.1001/archinte.168.9.920 18474755 PMC3093962

[B10] Baccarelli A Martinelli I Pegoraro V Melly S Grillo P Zanobetti A Hou L Bertazzi PA Mannucci PM Schwartz J Living near major traffic roads and risk of deep vein thrombosis Circulation 2009 119 3118 3124 10.1161/CIRCULATIONAHA.108.836163 19506111 PMC2895730

[B11] Dales RE Cakmak S Vidal CB Air Pollution and hospitalization for venous thromboembolic disease in Chile Journal of Thrombosis and Haemostasis 2010 **9999**. 10.1111/j.1538-7836.2010.03760.x 20088925

[B12] Kaplan GG Hubbard J Korzenik J Sands BE Panaccione R Ghosh S Wheeler AJ Villeneuve PJ The inflammatory bowel diseases and ambient air pollution: a novel association Am J Gastroenterol 2010 105 2412 2419 10.1038/ajg.2010.252 20588264 PMC3180712

[B13] Ananthakrishnan AN McGinley EL G BD Saeian K Air pollution and hospitalizations for inflammatory bowel disease: An ecologic analysis Gastroenterology 2010 138 S17 S18 10.1002/ibd.21455 20806342

[B14] Kaplan GG Dixon E Panaccione R Fong A Chen L Szyszkowicz M Wheeler A Maclean A Buie WD Leung T Heitman SJ Villeneuve PJ Effect of ambient air pollution on the incidence of appendicitis CMAJ 2009 10.1503/cmaj.082068 PMC2764754 19805497

[B15] Orazzo F Nespoli L Ito K Tassinari D Giardina D Funis M Cecchi A Trapani C Forgeschi G Vignini M Nosetti L Pigna S Zanobetti A Air pollution, aeroallergens, and emergency room visits for acute respiratory diseases and gastroenteric disorders among young children in six Italian cities Environ Health Perspect 2009 117 1780 1785 20049132 10.1289/ehp.0900599 PMC2801171

[B16] Guberan E Usel M Raymond L Bolay J Fioretta G Puissant J Increased risk for lung cancer and for cancer of the gastrointestinal tract among Geneva professional drivers Br J Ind Med 1992 49 337 344 1376139 10.1136/oem.49.5.337 PMC1039252

[B17] Gerhardsson de Verdier M Plato N Steineck G Peters JM Occupational exposures and cancer of the colon and rectum Am J Ind Med 1992 22 291 303 10.1002/ajim.4700220303 1519614

[B18] Andersen A Barlow L Engeland A Kjaerheim K Lynge E Pukkala E Work-related cancer in the Nordic countries Scand J Work Environ Health 1999 25 Suppl 2 1 116 10507118

[B19] Goldberg MS Parent ME Siemiatycki J Desy M Nadon L Richardson L Lakhani R Latreille B Valois MF A case-control study of the relationship between the risk of colon cancer in men and exposures to occupational agents Am J Ind Med 2001 39 531 546 10.1002/ajim.1052 11385637

[B20] Nel A Atmosphere. Air pollution-related illness: effects of particles Science 2005 308 804 806 10.1126/science.1108752 15879201

[B21] Semmler-Behnke M Takenaka S Fertsch S Wenk A Seitz J Mayer P Oberdorster G Kreyling WG Efficient elimination of inhaled nanoparticles from the alveolar region: evidence for interstitial uptake and subsequent reentrainment onto airways epithelium Environ Health Perspect 2007 115 728 733 10.1289/ehp.9685 17520060 PMC1867986

[B22] Urich D Soberanes S Burgess Z Chiarella SE Ghio AJ Ridge KM Kamp DW Chandel NS Mutlu GM Budinger GR Proapoptotic Noxa is required for particulate matter-induced cell death and lung inflammation FASEB J 2009 23 2055 2064 10.1096/fj.08-114546 19237507 PMC2704586

[B23] Ward NS Waxman AB Homer RJ Mantell LL Einarsson O Du Y Elias JA Interleukin-6-induced protection in hyperoxic acute lung injury Am J Respir Cell Mol Biol 2000 22 535 542 10783124 10.1165/ajrcmb.22.5.3808

[B24] Mutlu GM Budinger GR Green AA Urich D Soberanes S Chiarella SE Alheid GF McCrimmon DR Szleifer I Hersam MC Biocompatible nanoscale dispersion of single-walled carbon nanotubes minimizes in vivo pulmonary toxicity Nano Lett 2010 10 1664 1670 10.1021/nl9042483 20377197 PMC2869384

[B25] Budinger GR Mutlu GM Eisenbart J Fuller AC Bellmeyer AA Baker CM Wilson M Ridge K Barrett TA Lee VY Chandel NS Proapoptotic Bid is required for pulmonary fibrosis Proc Natl Acad Sci USA 2006 103 4604 4609 10.1073/pnas.0507604103 16537427 PMC1401229

[B26] Cani PD Possemiers S Van de Wiele T Guiot Y Everard A Rottier O Geurts L Naslain D Neyrinck A Lambert DM Muccioli GG Delzenne NM Changes in gut microbiota control inflammation in obese mice through a mechanism involving GLP-2-driven improvement of gut permeability Gut 2009 58 1091 1103 10.1136/gut.2008.165886 19240062 PMC2702831

[B27] Cani PD Bibiloni R Knauf C Waget A Neyrinck AM Delzenne NM Burcelin R Changes in gut microbiota control metabolic endotoxemia-induced inflammation in high-fat diet-induced obesity and diabetes in mice Diabetes 2008 57 1470 1481 10.2337/db07-1403 18305141

[B28] Erlejman AG Fraga CG Oteiza PI Procyanidins protect Caco-2 cells from bile acid- and oxidant-induced damage Free Radic Biol Med 2006 41 1247 1256 10.1016/j.freeradbiomed.2006.07.002 17015171

[B29] Buzza MS Netzel-Arnett S Shea-Donohue T Zhao A Lin CY List K Szabo R Fasano A Bugge TH Antalis TM Membrane-anchored serine protease matriptase regulates epithelial barrier formation and permeability in the intestine Proc Natl Acad Sci USA 2010 107 4200 4205 10.1073/pnas.0903923107 20142489 PMC2840089

[B30] Narai A Arai S Shimizu M Rapid decrease in transepithelial electrical resistance of human intestinal Caco-2 cell monolayers by cytotoxic membrane perturbents Toxicol In Vitro 1997 11 347 354 10.1016/S0887-2333(97)00026-X 20654321

[B31] Turner JR Angle JM Black ED Joyal JL Sacks DB Madara JL PKC-dependent regulation of transepithelial resistance: roles of MLC and MLC kinase Am J Physiol 1999 277 C554 562 10484342 10.1152/ajpcell.1999.277.3.C554

[B32] Turner JR Rill BK Carlson SL Carnes D Kerner R Mrsny RJ Madara JL Physiological regulation of epithelial tight junctions is associated with myosin light-chain phosphorylation Am J Physiol 1997 273 C1378 1385 9357784 10.1152/ajpcell.1997.273.4.C1378

[B33] Hubatsch I Ragnarsson EG Artursson P Determination of drug permeability and prediction of drug absorption in Caco-2 monolayers Nat Protoc 2007 2 2111 2119 10.1038/nprot.2007.303 17853866

[B34] Biganzoli E Cavenaghi LA Rossi R Brunati MC Nolli ML Use of a Caco-2 cell culture model for the characterization of intestinal absorption of antibiotics Farmaco 1999 54 594 599 10.1016/S0014-827X(99)00069-5 10555261

[B35] Banan A Zhang LJ Shaikh M Fields JZ Farhadi A Keshavarzian A Key role of PLC-gamma in EGF protection of epithelial barrier against iNOS upregulation and F-actin nitration and disassembly Am J Physiol Cell Physiol 2003 285 C977 993 12788694 10.1152/ajpcell.00121.2003

[B36] Banan A Zhang Y Losurdo J Keshavarzian A Carbonylation and disassembly of the F-actin cytoskeleton in oxidant induced barrier dysfunction and its prevention by epidermal growth factor and transforming growth factor alpha in a human colonic cell line Gut 2000 46 830 837 10.1136/gut.46.6.830 10807896 PMC1756435

[B37] Soberanes S Urich D Baker CM Burgess Z Chiarella SE Bell EL Ghio AJ De Vizcaya-Ruiz A Liu J Ridge KM Kamp DW Chandel NS Schumacker PT Mutlu GM Budinger GR Mitochondrial complex III-generated oxidants activate ASK1 and JNK to induce alveolar epithelial cell death following exposure to particulate matter air pollution J Biol Chem 2009 284 2176 2186 19033436 10.1074/jbc.M808844200 PMC2629089

[B38] Guzy RD Sharma B Bell E Chandel NS Schumacker PT Loss of the SdhB, but Not the SdhA, subunit of complex II triggers reactive oxygen species-dependent hypoxia-inducible factor activation and tumorigenesis Mol Cell Biol 2008 28 718 731 10.1128/MCB.01338-07 17967865 PMC2223429

[B39] Budinger GR Tso M McClintock DS Dean DA Sznajder JI Chandel NS Hyperoxia-induced apoptosis does not require mitochondrial reactive oxygen species and is regulated by Bcl-2 proteins J Biol Chem 2002 277 15654 15660 10.1074/jbc.M109317200 11877388

[B40] Chandel NS Schumacker PT Cells depleted of mitochondrial DNA (rho0) yield insight into physiological mechanisms FEBS Lett 1999 454 173 176 10.1016/S0014-5793(99)00783-8 10431801

[B41] King MP Attardi G Human cells lacking mtDNA: repopulation with exogenous mitochondria by complementation Science 1989 246 500 503 10.1126/science.2814477 2814477

[B42] Dooley CT Dore TM Hanson GT Jackson WC Remington SJ Tsien RY Imaging dynamic redox changes in mammalian cells with green fluorescent protein indicators J Biol Chem 2004 279 22284 22293 10.1074/jbc.M312847200 14985369

[B43] Hanson GT Aggeler R Oglesbee D Cannon M Capaldi RA Tsien RY Remington SJ Investigating mitochondrial redox potential with redox-sensitive green fluorescent protein indicators J Biol Chem 2004 279 13044 13053 14722062 10.1074/jbc.M312846200

[B44] Buccellato LJ Tso M Akinci OI Chandel NS Budinger GR Reactive oxygen species are required for hyperoxia-induced Bax activation and cell death in alveolar epithelial cells J Biol Chem 2004 279 6753 6760 14625274 10.1074/jbc.M310145200

[B45] Melov S Ravenscroft J Malik S Gill MS Walker DW Clayton PE Wallace DC Malfroy B Doctrow SR Lithgow GJ Extension of life-span with superoxide dismutase/catalase mimetics Science 2000 289 1567 1569 10.1126/science.289.5484.1567 10968795

[B46] Banan A Zhang LJ Shaikh M Fields JZ Farhadi A Keshavarzian A Novel effect of NF-kappaB activation: carbonylation and nitration injury to cytoskeleton and disruption of monolayer barrier in intestinal epithelium Am J Physiol Cell Physiol 2004 287 C1139 1151 10.1152/ajpcell.00146.2004 15175222

[B47] Banan A Zhang LJ Farhadi A Fields JZ Shaikh M Forsyth CB Choudhary S Keshavarzian A Critical role of the atypical {lambda} isoform of protein kinase C (PKC-{lambda}) in oxidant-induced disruption of the microtubule cytoskeleton and barrier function of intestinal epithelium J Pharmacol Exp Ther 2005 312 458 471 15347733 10.1124/jpet.104.074591

[B48] Banan A Zhang LJ Farhadi A Fields JZ Shaikh M Keshavarzian A PKC-beta1 isoform activation is required for EGF-induced NF-kappaB inactivation and IkappaBalpha stabilization and protection of F-actin assembly and barrier function in enterocyte monolayers Am J Physiol Cell Physiol 2004 286 C723 738 10.1152/ajpcell.00329.2003 14602581

[B49] Renard P Ernest I Houbion A Art M Le Calvez H Raes M Remacle J Development of a sensitive multi-well colorimetric assay for active NFkappaB Nucleic Acids Res 2001 29 E21 10.1093/nar/29.4.e21 11160941 PMC29628

[B50] Banan A Choudhary S Zhang Y Fields JZ Keshavarzian A Ethanol-induced barrier dysfunction and its prevention by growth factors in human intestinal monolayers: evidence for oxidative and cytoskeletal mechanisms J Pharmacol Exp Ther 1999 291 1075 1085 10565827

[B51] Mutlu GM Snyder C Bellmeyer A Wang H Hawkins K Soberanes S Welch LC Ghio AJ Chandel NS Kamp D Sznajder JI Budinger GR Airborne particulate matter inhibits alveolar fluid reabsorption in mice via oxidant generation Am J Respir Cell Mol Biol 2006 34 670 676 10.1165/rcmb.2005-0329OC 16439801 PMC2644228

[B52] Barnes PJ Karin M Nuclear factor-kappaB: a pivotal transcription factor in chronic inflammatory diseases N Engl J Med 1997 336 1066 1071 10.1056/NEJM199704103361506 9091804

[B53] Mutlu E Keshavarzian A Engen P Forsyth CB Sikaroodi M Gillevet P Intestinal dysbiosis: a possible mechanism of alcohol-induced endotoxemia and alcoholic steatohepatitis in rats Alcohol Clin Exp Res 2009 33 1836 1846 10.1111/j.1530-0277.2009.01022.x 19645728 PMC3684271

[B54] Brook RD Franklin B Cascio W Hong Y Howard G Lipsett M Luepker R Mittleman M Samet J Smith SC Jr Tager I Air pollution and cardiovascular disease: a statement for healthcare professionals from the Expert Panel on Population and Prevention Science of the American Heart Association Circulation 2004 109 2655 2671 10.1161/01.CIR.0000128587.30041.C8 15173049

[B55] Mutlu GM Green D Bellmeyer A Baker CM Burgess Z Rajamannan N Christman JW Foiles N Kamp DW Ghio AJ Chandel NS Dean DA Sznajder JI Budinger GR Ambient particulate matter accelerates coagulation via an IL-6-dependent pathway J Clin Invest 2007 117 2952 2961 10.1172/JCI30639 17885684 PMC1978421

[B56] Peters A von Klot S Heier M Trentinaglia I Hormann A Wichmann HE Lowel H Exposure to traffic and the onset of myocardial infarction N Engl J Med 2004 351 1721 1730 10.1056/NEJMoa040203 15496621

[B57] McKenzie SJ Baker MS Buffinton GD Doe WF Evidence of oxidant-induced injury to epithelial cells during inflammatory bowel disease J Clin Invest 1996 98 136 141 10.1172/JCI118757 8690784 PMC507409

[B58] Banan A Farhadi A Fields JZ Mutlu E Zhang L Keshavarzian A Evidence that nuclear factor-kappa B activation is critical in oxidant-induced disruption of the microtubule cytoskeleton and barrier integrity and that its inactivation is essential in epidermal growth factor-mediated protection of the monolayers of intestinal epithelia J Pharmacol Exp Ther 2003 306 13 28 10.1124/jpet.102.047415 12815011

[B59] Rogler G Brand K Vogl D Page S Hofmeister R Andus T Knuechel R Baeuerle PA Scholmerich J Gross V Nuclear factor kappaB is activated in macrophages and epithelial cells of inflamed intestinal mucosa Gastroenterology 1998 115 357 369 10.1016/S0016-5085(98)70202-1 9679041

[B60] Schreiber S Nikolaus S Hampe J Activation of nuclear factor kappa B inflammatory bowel disease Gut 1998 42 477 484 10.1136/gut.42.4.477 9616307 PMC1727068

[B61] Keshavarzian A Banan A Farhadi A Komanduri S Mutlu E Zhang Y Fields JZ Increases in free radicals and cytoskeletal protein oxidation and nitration in the colon of patients with inflammatory bowel disease Gut 2003 52 720 728 10.1136/gut.52.5.720 12692059 PMC1773652

[B62] Lammers KM Lu R Brownley J Lu B Gerard C Thomas K Rallabhandi P Shea-Donohue T Tamiz A Alkan S Netzel-Arnett S Antalis T Vogel SN Fasano A Gliadin induces an increase in intestinal permeability and zonulin release by binding to the chemokine receptor CXCR3 Gastroenterology 2008 135 194 204 e193 10.1053/j.gastro.2008.03.023 18485912 PMC2653457

[B63] Kumagai Y Arimoto T Shinyashiki M Shimojo N Nakai Y Yoshikawa T Sagai M Generation of reactive oxygen species during interaction of diesel exhaust particle components with NADPH-cytochrome P450 reductase and involvement of the bioactivation in the DNA damage Free Radic Biol Med 1997 22 479 487 10.1016/S0891-5849(96)00341-3 8981040

[B64] Xia T Korge P Weiss JN Li N Venkatesen MI Sioutas C Nel A Quinones and aromatic chemical compounds in particulate matter induce mitochondrial dysfunction: implications for ultrafine particle toxicity Environ Health Perspect 2004 112 1347 1358 10.1289/ehp.7167 15471724 PMC1247559

[B65] Hiura TS Kaszubowski MP Li N Nel AE Chemicals in diesel exhaust particles generate reactive oxygen radicals and induce apoptosis in macrophages J Immunol 1999 163 5582 5591 10553087

[B66] Karczewski JM Peters JG Noordhoek J Prevention of oxidant-induced cell death in Caco-2 colon carcinoma cells after inhibition of poly(ADP-ribose) polymerase and Ca2+ chelation: involvement of a common mechanism Biochem Pharmacol 1999 57 19 26 10.1016/S0006-2952(98)00286-X 9920281

[B67] Ferruzza S Scacchi M Scarino ML Sambuy Y Iron and copper alter tight junction permeability in human intestinal Caco-2 cells by distinct mechanisms Toxicol In Vitro 2002 16 399 404 10.1016/S0887-2333(02)00020-6 12110278

[B68] Mage D Ozolins G Peterson P Webster A Orthofer RVV Gwynne M Urban air pollution in megacities of the world Atmospheric Environment 1996 30 681 686 10.1016/1352-2310(95)00219-7

[B69] Benchmarking Urban Air Quality Management and Practice in Major and Mega Cities of Asia http://www.unep.org/PDF/APMA_Benchmarking_report.pdf

[B70] de Hennezel L Debarre S Ramisse F Delamanche S Harf A Alonso JM Calvet JH Plethysmography for the assessment of pneumococcal pneumonia and passive immunotherapy in a mouse model Eur Respir J 2001 17 94 99 10.1183/09031936.01.17100940 11307763

